# Inventory construction to track cognitive profiles compatible with intellectual disability, ADHD, and dyslexia in children between 6 to 11 years old

**DOI:** 10.1192/j.eurpsy.2021.2001

**Published:** 2021-08-13

**Authors:** L.R. Carreiro, R.L. Marino, A. Souza

**Affiliations:** Developmental Disorders Graduate Program, Mackenzie Presbyterian University, São Paulo, Brazil

**Keywords:** Screening Inventory, ADHD, Dyslexia, intellectual disability

## Abstract

**Introduction:**

The most frequent complaints from children referred to psychiatry and psychologist are related to academic and attentional impairments, or developmental delay, which are shared by many conditions.

**Objectives:**

To develop an inventory that evaluates cognitive functions of children between 6 to 11 years old to track cognitive profiles compatible with Intellectual Disability, ADHD, and dyslexia to assist in differential diagnosis.

**Methods:**

In study 1 (identification of the relevant constructs, operational definition, and items development), data were collected from parents of children and professionals who serve this population; articles, verification of screening instruments and identification of cognitive impairments by the DSM-5. In study 2, an analysis of judges with professionals was carried out, as well as a verification of the items’ clarity by the target population; In study 3, we looked for evidence of validity and precision indicators with a sample of 272 parents and 178 teachers of 72 children diagnosed with one of the three disorders and 207 that had no suspect of neurodevelopmental disorders.

**Results:**

For the parent version, the four-factor solution was the most appropriate, with the following Scales: Attention, Executive Functions, Intelligence and Oral Language. The final version for parents was composed of 60 items, with excellent internal consistency indices (coefficients> 0.90).

**Conclusions:**

ROC curves expressed good sensitivity and specificity of the scales for each disorder. Future studies have to expand the sample size of children diagnosed with one of the three disorders so that new analyzes can be performed and the results can be generalizable to the population.

**Disclosure:**

No significant relationships.

